# Study protocol: Type III hybrid effectiveness-implementation study implementing Age-Friendly evidence-based practices in the VA to improve outcomes in older adults

**DOI:** 10.1186/s43058-023-00431-5

**Published:** 2023-05-25

**Authors:** Kirstin Manges Piazza, Laura Ellen Ashcraft, Liam Rose, Daniel E. Hall, Rebecca T. Brown, Mary Elizabeth (Libbey) Bowen, Shahrzad Mavandadi, Alison C. Brecher, Shimrit Keddem, Bruce Kiosian, Judith A. Long, Rachel M. Werner, Robert E. Burke

**Affiliations:** 1 Center for Health Equity Research and Promotion, Corporal Michael J. Crescenz VA Medical Center, 3900 Woodland Ave, Philadelphia, PA USA; 2 Division of General Internal Medicine, Department of Medicine, University of Pennsylvania Perelman School of Medicine, Philadelphia, PA USA; 3 Stanford-Surgery Policy Improvement Research & Education Center, Stanford University, Stanford, CA USA; 4 Health Economics Resource Center, VA Palo Alto Health Care System, Palo Alto, CA USA; 5 Center for Health Equity Research and Promotion, VA Pittsburgh Healthcare System, Pittsburgh, PA USA; 6 Geriatric Research Education and Clinical Center, VA Pittsburgh Healthcare System, Pittsburgh, PA USA; 7 Department of Surgery, University of Pittsburgh, Pittsburgh, PA USA; 8 Wolff Center, University of Pittsburgh Medical Center, Pittsburgh, PA USA; 9 Geriatrics and Extended Care Program, Corporal Michael Crescenz VA Medical Center, Philadelphia, PA USA; 10 Education, and Clinical Center, VISN4 Mental Illness Research, Corporal Michael JCrescenz VA Medical Center, Philadelphia, PA USA; 11 School of Nursing, University of Delaware, Newark, DE USA; 12 Thomas Jefferson University, Philadelphia, PA USA; 13 Leonard Davis Institute of Health Economics, University of Pennsylvania, Philadelphia, PA USA; 14 Department of Family Medicine & Community Health, University of Pennsylvania Perelman School of Medicine, Philadelphia, PA USA

**Keywords:** Implementation science, Veterans Health Administration, Veterans, Age-friendly, Geriatrics, Age-friendly healthcare system

## Abstract

**Background:**

Unmet care needs among older adults accelerate cognitive and functional decline and increase medical harms, leading to poorer quality of life, more frequent hospitalizations, and premature nursing home admission. The Department of Veterans Affairs (VA) is invested in becoming an “Age-Friendly Health System” to better address four tenets associated with reduced harm and improved outcomes among the 4 million Veterans aged 65 and over receiving VA care. These four tenets focus on “4Ms” that are fundamental to the care of older adults, including (1) what *M*atters (ensuring that care is consistent with each person’s goals and preferences); (2) *M*edications (only using necessary medications and ensuring that they do not interfere with what matters, mobility, or mentation); (3) *M*entation (preventing, identifying, treating, and managing dementia, depression, and delirium); and (4) *M*obility (promoting safe movement to maintain function and independence). The Safer Aging through Geriatrics-Informed Evidence-Based Practices (SAGE) Quality Enhancement Research Initiative (QUERI) seeks to implement four evidence-based practices (EBPs) that have shown efficacy in addressing these core tenets of an “Age-Friendly Health System,” leading to reduced harm and improved outcomes in older adults.

**Methods:**

We will implement four EBPs in 9 VA medical centers and associated outpatient clinics using a type III hybrid effectiveness-implementation stepped-wedge trial design. We selected four EBPs that align with Age-Friendly Health System principles: Surgical Pause, EMPOWER (Eliminating Medications Through Patient Ownership of End Results), TAP (Tailored Activities Program), and CAPABLE (Community Aging in Place – Advancing Better Living for Elders). Guided by the Pragmatic Robust Implementation and Sustainability Model (PRISM), we are comparing implementation as usual vs. active facilitation. Reach is our primary implementation outcome, while “facility-free days” is our primary effectiveness outcome across evidence-based practice interventions.

**Discussion:**

To our knowledge, this is the first large-scale randomized effort to implement “Age-Friendly” aligned evidence-based practices. Understanding the barriers and facilitators to implementing these evidence-based practices is essential to successfully help shift current healthcare systems to become Age-Friendly. Effective implementation of this project will improve the care and outcomes of older Veterans and help them age safely within their communities.

**Trial registration:**

Registered 05 May 2021, at ISRCTN #60,657,985.

**Reporting guidelines:**

Standards for Reporting Implementation Studies (see attached).

**Supplementary Information:**

The online version contains supplementary material available at 10.1186/s43058-023-00431-5.

Contribution to the literature

- Veterans aged 65 and older who do not receive age-friendly care are at increased risk for poor outcomes.
- The Age-Friendly Health System model of What Matters, Medications, Mentation, and Mobility (4Ms) is an evidence-based framework for ensuring older adults receive holistic care that aligns with their wishes.
- Studies have not evaluated the adoption of 4Ms care on a large scale within a national health care system
- This study will evaluate the implementation of four EBPs across nine VA medical centers to assess EBP “Reach” and facility-free days.

## Background

Older adults have needs that are not being met by the current health care system [1, 2]. As medical complexity and functional impairment among older populations increase, health systems increasingly struggle to provide high-quality, goal-concordant care [1–3]. Inappropriate care and unmet care needs among older adults accelerate cognitive and functional decline, leading to poorer quality of life, more frequent hospital utilization, and premature nursing home admission [1, 2]. Recognizing persistent quality gaps in care for older adults nationally, the Institute for Healthcare Improvement (IHI) and The John A. Hartford Foundation are leading a national campaign to make health systems “Age Friendly” [3–6].

**Fig. 1:**
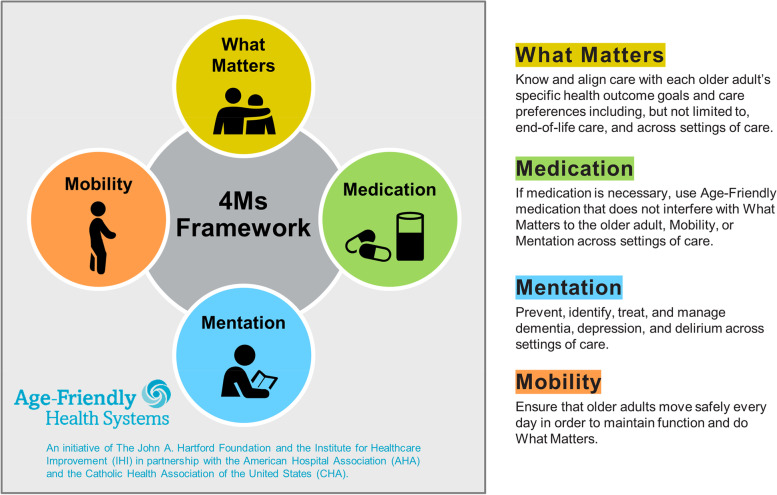
The Institute for Healthcare Improvement model of Age-Friendly Health Systems

The Age-Friendly Health System (AFHS) model is a patient-centered framework that incorporates evidence-based elements of high-quality care by focusing on “4Ms” that are fundamental to the care of older adults (Fig. 1). These include (1) what *M*atters (ensuring that care is consistent with each person’s goals and preferences); (2) *M*edications (only using necessary medications and ensuring that they do not interfere with what matters, mobility, or mentation); (3) *M*entation (preventing, identifying, treating, and managing dementia, depression, and delirium); and (4) *M*obility (promoting safe movement to maintain function and independence) [1, 2, 4–6]. For this model to be successful, there is a critical need to understand how to implement and sustain AFHS at scale [5]. To date, the evidence regarding AFHS implementation best practices is limited to case studies [7, 8]. Furthermore, it is unclear which of the 4 M-concordant interventions are most impactful for the outcomes of older adults who dwelling in the community [3, 4, 9–12].

With more than 4 million Veterans aged 65 and older [13], the VA is a promising context for implementation of the AFHS model. The Safer Aging through Geriatrics-Informed Evidence-Based Practices (SAGE) Quality Enhancement Research Initiative (QUERI) program, funded by VA QUERI, is intended to address these gaps. This program aims to test different implementation strategies while implementing four evidence-based practices (each aligned with one of the 4Ms) across nine VA medical centers and their associated community-based outpatient clinics with in the Veteran Integrated Service Network (VISN) 4 (*see map—*https://www.visn4.va.gov/VISN4/locations/map.html).

### Study goals and objectives

Our goals in the SAGE QUERI are threefold: (1) to compare different implementation strategies in a randomized fashion to understand how the interaction between implementation strategies, local site context, and intervention characteristics lead to different implementation outcomes; (2) to measure the impact of the implementation of evidence-based practices (or EBPs) on outcomes of older Veterans who are community dwelling; and (3) understand how these EBPs can most successfully be adapted for the VA context to allow successful national dissemination and maintenance in clinical practice.

### Conceptual framework and theoretical foundation

**Fig. 2:**
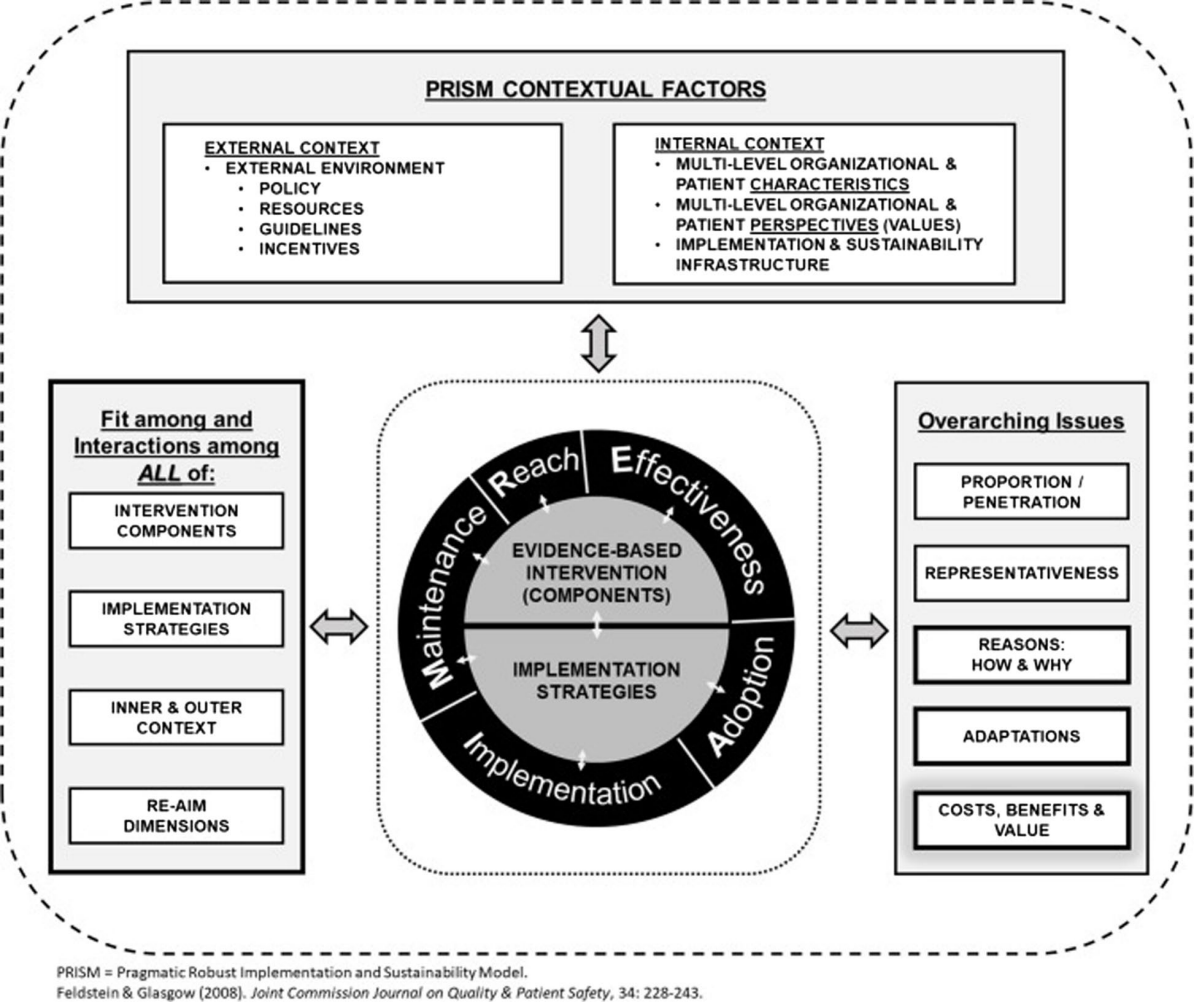
The Pragmatic Robust Implementation and Sustainability Model [14]

Our approach builds on the AFHS conceptual framework supported by IHI and The John A. Hartford Foundation [3, 6]. We are employing the Practical, Robust Implementation and Sustainability Model (PRISM) as a theoretical foundation to guide our pre-implementation assessment and implementation evaluation (Table 1 and Fig. 2; [14]). PRISM draws upon and integrates key concepts from Diffusion of Innovations Theory, IHI quality improvement models, and the Chronic Care Model [14, 15]. PRISM is an extension of the original Reach Effectiveness-Adoption Implementation Maintenance (RE-AIM) evaluation model [14, 15].

**Table 1:** Assessing PRISM domains to understand context for implementation

PRISM domain	What we are assessing	Data collection techniques
Organization perspectives and values	- Staff perspectives evidence base - Staff perspectives usefulness in local context - Current workflow processes - How each EBP fits into the broader organization - Potential contextual factors that may facilitate/limit implementation - Job satisfaction	- Key informant interviews - Site visit rapid ethnography observations - Staff process mapping - Brainwriting activity - VA all employee surveys (for example SAIL ratings)
Patient perspective and values	- Veteran perspectives on key intervention components - Overall program satisfaction with care - Identify potential unmet needs	- Site visit rapid ethnography observations - Veteran Advisory Board feedback - SAIL metrics
Organizational recipient characteristics	- Existing quality gaps documented by data and as perceived by staff - Organizational priorities as perceived by frontline staff and leaders - Staffing and turnover - Interfacility communication - Existing quality improvement initiatives	- National level VA quantitative data - VA all employee surveys - Key informant interviews - Brainwriting activity
Patient recipient characteristics	- Sociodemographic characteristics - Health status - Competing programs or demands on patients - Stories of patients who could be impacted by AFHS - Overall program satisfaction with care	- National level VA quantitative data - Site visit rapid ethnography observations - Veteran Advisory Board feedback
Implementation and sustainability infrastructure	- Existing processes and systems - Prior experience with new initiatives - Culture regarding satisfaction with work and new initiatives - Relationships between key stakeholders - Resources for implementation and sustainment - Staff readiness to change	- Key informant interviews - Brainwriting activity - Staff process mapping
External environment	- Current regulatory environment as perceived by staff/leaders - Changes in organization due to external changes (MISSION, changing patient population, reimbursement) - Existing guidelines or incentives	- Key informant interviews - Brainwriting activity - Review of artifacts - VA all employee surveys - National level VA quantitative data

PRISM provides a useful framework for assessing implementation barriers and facilitators and has been used to evaluate multiple VA and non-VA health systems interventions [15–21]. Attention to PRISM “Contextual Factors” are being used to focus and organize pre-implementation site assessment on specific domains (Fig. 2 and Table 1). These contextual factors include internal factors (e.g., multiple level organizational patient characteristics, multiple level organizational patient perspectives, implementation and sustainability infrastructure) and external factors (e.g., policy, resources, guidelines, incentives). To tailor the implementation strategies at each site, the concept of “Fit” (Fig. 2) will guide proactive planning of what was learned in the pre-implementation assessment—such as attention to inner and outer context—to what is known about the intervention components.

The PRISM model also highlights the key interplay between implementation strategies and EBPs and calls for ongoing evaluation (using RE-AIM) to generate feedback during the study. We operationalize RE-AIM (Table 2) as the following: *Reach* (proportion of eligible population receiving each EBP), *Effectiveness* (evidence of effects on health outcomes), *Adoption* (proportion of clinical sites or teams implementing the EBP), *Implementation* (fidelity to the EBP and adaptations made), and *Maintenance* (what proportion of sites continue to use the EBPs after active implementation ceases). Additionally, we plan incorporate “Overarching Issues” (Fig. 2) to better understand “how” and “why” implementation did or did not work. RE-AIM emphasizes the importance of tracking adaptations, identifying the representativeness of the sample, and evaluating costs and benefits [22]. This framework informs the entire implementation process.

**Table 2:** Reach, Effectiveness, Adoption, Implementation, Maintenance (RE-AIM) outcome

Construct	Definition [22]	SAGE outcomes
Reach	The number or proportion of people who participate in the intervention	Proportion of eligible Veterans who receive each EBP
Effectiveness	The effect of the intervention on relevant outcomes (e.g., quality of life, economic outcomes)	Facility-free days: the number of days an older Veteran remains alive and outside the hospital or nursing home
Adoption^a^	The number or proportion of sites or individuals who are willing to initiate the program	Proportion of VA Medical Center (or service line) who begin implementation of the EBP
Implementation^a^	Fidelity to the core components of the intervention	Of adopting sites, proportion of EBP interactions that complete 80% or more of EBP core components
Maintenance^a^	Sustainment of the program or behavior over a period of 6 months	Of adopting sites, proportion of eligible Veterans enrolled in EBP is stable at 1 year follow-up

^a^SAGE QUERI secondary outcomes

## Methods and design

### Overview of study design

To achieve our goals, we are conducting a randomized, type III hybrid effectiveness-implementation study using a stepped-wedge design to compare passive implementation or “implementation as usual” to facilitation as the primary implementation strategy [23, 24]. We selected facilitation as the primary implementation strategy because each EBP is multi-component and none have fully been used before in the VA, potentially necessitating a higher-intensity and flexible strategy [25, 26]. However, given the anticipated spread of the AFHS model in the VA, we decided to contrast this strategy with implementation “as usual,” which is less intensive and costly and more aligned with IHI’s model. The SAGE QUERI was designed for internal VA operational proposes. In January 2019, all planned procedures were determined by the VISN Chief Medical Officer to be operations activities not constituting research and proceeded as such under VISN authority and oversight without IRB review according to the provision of VHA Program Guide 1200.21.

Our primary effectiveness outcome is “facility-free days,” generally defined as days alive and outside a hospital or nursing home [27–29]. Our primary implementation outcome is Reach, defined as how many eligible Veterans receive each intervention. Unlike typical cluster trials that assign clusters of sites to a control/comparison condition or an active treatment condition, all sites in a stepped wedge design eventually receive the active treatment condition after receiving the comparison condition for a scheduled period [23, 24]. This design is ethically indicated in circumstances when equipoise is insufficient to justify the use of a control condition for the entire study period (e.g., when the principle of justice precludes withholding an intervention proven beneficial). Clusters are randomized to start time for the more active implementation phase. As shown in the Additional file 1: Figure S1, we plan to use three “steps” with each including three VA medical centers and their associated clinics, randomizing each cluster to a different start time for Active Implementation.

### Description of interventions

We conducted a literature review of published interventions within each of the 4Ms to select an EBPs for implementation. We limited our search to interventions with either existing randomized, controlled trials, a strong evidence of clinical impact, and/or a history of successful prior implementation in the VA. We identified interventions that could be implemented in different care settings within each clinical site, as we hypothesized that asking a single care setting to initiate and support four different EBPs at once would not be feasible. Finally, we compared published interventions to existing VA priorities and programs with the help of VA national leaders to identify areas of alignment. As shown in Table 3, we selected four EBPs: Surgical Pause, EMPOWER (Eliminating Medications Through Patient Ownership of End Results), TAP (Tailored Activities Program), and CAPABLE (Community Aging in Place – Advancing Better Living for Elders).

**Table 3:** Summary of age-friendly evidence-based practice interventions

	Surgical pause	Empower	TAP	CAPABLE
Description	“Surgical Pause” involves systematic screening for frailty using the Risk Analysis Index (RAI) prior to surgery, triggering care pathways that clarify goals pre-operatively	“Eliminating Medications through Patient Ownership of End Results” (EMPOWER) is a direct-to-consumer booklet sent to older adults receiving a high-risk medication on a chronic basis paired with academic detailing for prescribers	“Tailored Activities Program” (TAP) is a 4-month structured, manualized intervention led by an occupational therapist (OT) that trains caregivers of patients with dementia to deliver activities tailored to the care recipients’ interests and capabilities	“Community Aging in Place – Advancing Better Living for Elders” (CAPABLE) utilizes a nurse, occupational therapist, and handyperson to identify and facilitate the functional goals related to aging in place of low-income older adults with 1 ADL or 2 IADL impairments
Core components	• Prior to surgical decision making, patient completes the RAI • Patients with RAI ≥ 37 referred for goal clarification conversation • Clinician(s) and patient review treatment plan, with or without surgery, to align with patient’s overarching goals and values	• Identifying patient panel • Mail EMPOWER brochure which includes a self-assessment of risks for medication, presentation of evidence of harms, medication education, and suggestions for conversation with provider • Provider prescriber education	• OT formal training • OT conducts home assessment to identify Veterans’ preserved capabilities and interests • OT develops 3 tailored activity prescriptions • Caregiver and Veteran participate in at least 3 TAP sessions	• OT and nurse formal training • Staffing components: • OT assess functional abilities, home accessibility, and safety then collaborates with Veteran to create up to 3 ADL/IDAL goals • Nurse—assesses medical status and develops 3 nursing goals • Handy Person—completes home modifications/repairs
Example adaptations	• Which staff screen • Strategy for communicating results to surgeon • In-person versus virtual training • Which clinician completes goal clarification conversation	• Format of brochure • Timing of brochure mailing and communication with prescriber • Which staff mail the brochure • Number of mailings	• Other health professionals (social worker, psychologist, RN) may also implement TAP with OT supervision • Location of session • Number of visits	• Referral source • Addressing more critical and expensive home repairs • Number of sessions conducted virtually vs in home after the initial assessment
Patient eligibility	• Age 65 + • RAI ≥ 37 • Considering “major surgery” as defined by a VASQIP-eligible CPT code	• Age 65 + • Community-dwelling (i.e., not residing in a nursing home) • Receiving a prescription for a targeted drug class for at least 90 of the last 120 days from a VA prescriber	• Age 65 + • Has a dementia diagnosis • Has an adult caregiver willing/able to participate • Can participate in at least two basic ADLs	• Age 65 + • Low income • At least 1 ADL or 2 IADL deficiencies • Able to ambulate around house independently with or without an assistive device
Potential setting	Preoperative clinics (e.g., surgery, anesthesia, primary care, medical subspecialties)	Primary care, mental health	Home care, home-based primary care, caregiver support program	Home care

*VASQIP* Veterans Affairs Surgical Quality Improvement Program, *CPT code* Current Procedural Terminology, *VA* Veterans Administration, *RN* registered nurse, *IADL* Instrumental Activities of Daily Living

Each EBP has a primary focus on one of the 4Ms, yet all incorporate multiple age-friendly domains. The Surgical Pause (What Matters) is a preoperative frailty screening program that triggers referral of frail patients for a structured goal clarification conversation to ensure surgical treatment aligns with patient priorities [30–32]. EMPOWER (Medications) is a direct-to-consumer intervention shown to more than triple the rate of discontinuation of high-risk medications among older adults [33–37]. TAP (Mentation) is a home-based intervention that reduces functional dependence, dementia-related symptoms, and caregiver burden [38–41]. CAPABLE (Mobility) is a multidisciplinary home-based intervention that provides nursing care, occupational therapy, and home adaptations for older adults with impairments in Instrumental and Activities of Daily Living [42–44]. These EBPs are not mandated or part of routine clinical care both in VA and non-VA settings, despite substantial evidentiary support.

### Study context

This project is occurring in VISN 4, a demographically and geographically diverse region comprising 9 VA Medical Centers covering 83 counties in Pennsylvania and Delaware and parts of Ohio, West Virginia, New York, and New Jersey. In VISN 4, 62% of the approximately 275,000 enrolled Veterans who use the VA for care are 65 or older; in 2018, 16% of this cohort was hospitalized at least once and 7% entered a nursing home for long-term care. These rates vary significantly across VISN 4 Medical Centers (11–24% and 4–8%, respectively) and are much higher in specific vulnerable populations. For example, Veterans with dementia had a 45% hospitalization rate and 20% newly entered nursing homes in 2018. The baseline variation in these rates suggests a potential opportunity to improve care of older Veterans who wish to avoid the hospital and “age in place.” Notably, the VA Medical Centers in VISN 4 include larger urban academic hospital campuses which provide more complex care (e.g., Pittsburgh, Philadelphia) as well as many rural sites (e.g., Erie, Altoona). The diversity of populations in VISN 4 will provide insight into potentially unique contextual implementation differences.

### Target sites

All nine VA Medical Centers (VAMC) in VISN 4 and their associated outpatient clinics will be eligible to participate in the four EBPs to the degree appropriate for their site. For example, every site has pharmacists (which is critical to EMPOWER), and all nine VA Medical Centers have Home-Based Primary Care (HBPC) or home care agencies suitable for implementation of TAP and CAPABLE—but only five sites across VISN 4 perform intermediate or complex surgeries suitable for the Surgical Pause. For TAP, CAPABLE, and EMPOWER, we are randomizing at the VAMC-level, and for Surgical Pause, we are randomizing at the level of the surgical service line level to achieve balance between the 3 clusters regarding case volume, case complexity, and patient comorbidity. Each medical center can decide which EBPs to participate in, although we are encouraging medical centers to adopt all four, where applicable, adhering to the AFHS model.

### Target population

Veterans eligible for inclusion in SAGE must be age 65 or older, community-dwelling (not in a long-term nursing facility for more than 100 days prior to receiving an EBP), and either receiving a treatment targeted by one of our EBPs (e.g., prescribed a high-risk medication or evaluated by a surgeon in consideration for a possible surgical procedure) or have a risk factor targeted by one of our EBPs (e.g., diagnosis of dementia, or functional impairments that prevent completion of at least one Activities of Daily Living [ADL] and are low-income). Each EBP has additional eligibility criteria that mirror criteria from the trials supporting each EBP (Table 3). Exclusion criteria for all EBPs include current receipt of hospice or current residence in long-term nursing home care. In addition, Veterans with severe mental illness will be excluded from EMPOWER, and Veterans without a caregiver will be excluded from TAP.

### Partnership approach

#### Evaluation team

The SAGE evaluation team is multidisciplinary and composed of experts in implementation science, mixed methods research, health systems research, health economics, quality improvement, data management, nursing, medicine, surgery, geriatrics, social work, psychology, gerontology, occupational therapy, and hospital administration.

#### Operational partners

QUERI program centers are required to demonstrate substantial a priori operational support to enhance the likelihood of successful project completion. In the case of SAGE QUERI, our key partners include the VA national office of Geriatrics and Extended Care and the Geriatrics and Extended Care Data Analysis Center (GEC DAC), the VA national Surgical Office, and VISN 4 leadership including the Director and Chief Medical Officer, as well as individual VAMC Directors and Chiefs of Staff. SAGE QUERI will assemble a VISN-wide Veterans Community Advisory Board to engage Veterans in implementation and dissemination activities. Further, we have enlisted multiple advisors for the project to serve on a Technical Expert Panel, including representatives from the IHI, geriatrics leaders, and VA administrators. The evaluation team is independent of the operational partners while simultaneously receiving feedback and guidance on mutual goals regarding implementation of the four EBPs.

## Study phases

**Fig. 3:**
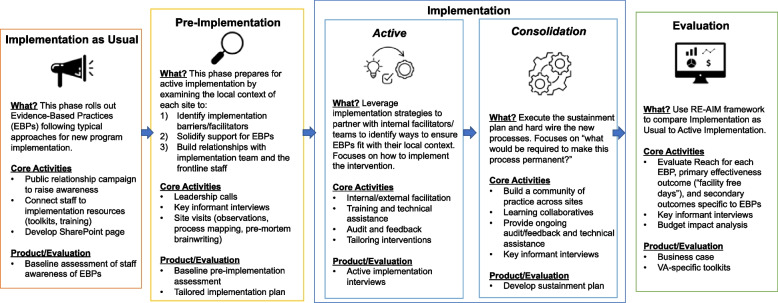
Summary of Safer Aging Through Geriatrics-Informed Evidence-Based Practices project phases

As illustrated in Fig. 3 and described below, each site will move through five consecutive phases: (1) implementation as usual, (2) pre-implementation, (3) active implementation, (4) consolidation, and (5) evaluation.

### Phase 1: Implementation as Usual (2–20 months depending on site)

Each cluster of sites will begin with an “Implementation as Usual” phase, which will involve rolling out EBPs in the manner that is typical for new program implementation in the VA. Implementation as Usual includes a public relations campaign to raise awareness of the SAGE program and EBPs and connects leadership and front-line staff to EBP-related resources. Dissemination approaches will include using social media (Twitter, VA Pulse), hosting a VISN SharePoint page, sending targeted emails using GovDelivery, and using digital bulletin boards and infographics placed in clinical areas. These communications are commonly used approaches by the VA and similar organizations such as the Agency for Health Research and Quality and the Centers for Disease Control and Prevention [45, 46]. This phase will allow us to capture baseline data (e.g., number of staff accessing the EBP materials), including answering qualitative questions (e.g., “Have you hear about SAGE QUERI? If so, how?”), and allow for measurement of any incremental benefit of the more active (intense) implementation approach in phase 3.

### Phase 2: Pre-Implementation (6–7 months)

Each cluster of sites will undergo a “Pre-Implementation” phase to accomplish three goals: (1) identify barriers and facilitators to implementation, (2) solidify front-line staff support for implementing the EBPs, and (3) build relationships between the implementation team and front-line staff that promote Adoption and Maintenance of the EBPs. Our pre-implementation contextual inquiry will include conducting rapid content analysis of key informant interviews and site visits (e.g., observations, group process mapping, and group pre-mortem brainstorming). Data we collect during the pre-implementation phase will be used to tailor the implementation process at each site during the implementation phase and as baseline data for evaluation.

#### Key informant interviews

To better understand the local context and map processes, we are conducting semi-structured key informant stakeholder interviews with staff virtually at each site. We will use a purposive convenience snowball sampling approach to interview approximately 5 to 10 administrators and clinicians individually per site for each EBP. We are using semi-structured interview guides and parallel memo templates for each EBP based on the PRISM domains (Table 3). For example, questions explore staff’s knowledge and impressions of the EBP, understanding of current practices related to key processes important to each EBP (such as deprescribing for EMPOWER or enrolling patients in caregiver support programs for TAP), perceptions of how the EBP could be integrated into current practice, and general views regarding potential facilitators and barriers to implementation. These interviews are anticipated to last approximately 30 to 60 min, will be audio-recorded, and transcribed for analysis.

#### Site visits

Following the completion of key stakeholder interviews, we are conducting site visits (either in person or virtually) which include informational sessions with leadership, observations of current processes, and group activities with frontline staff. To help garner support, the leadership informational sessions will provide an overview of the EBPs, present historical site-specific quality and performance data related to the EBPs, and provide an opportunity to answer questions. At each site per EBP, trained SAGE staff will conduct observations of key settings and/or staff members to gain an understanding of the context, processes, and interpersonal dynamics of the clinical sites. Informed by rapid ethnographic approaches [23, 47, 48] and the PRISM framework (Table 1), we will use structured tools and unstructured fieldnotes to record observational data.

Additionally, for each EBP, we will use two group activities involving novel rapid site assessment tools informed by PRISM domains—a brainwriting premortem exercise [49] and a process mapping exercise [50, 51]—to further understand the perspectives of frontline staff. The “brainwriting premortem” is a focus group technique that uses silent sharing of written ideas about how a proposed implementation process will fail; it allows stakeholders to express concerns and think through potential barriers in advance. Participants are then invited to brainstorm solutions to the perceived barriers and anticipated failure points [49, 50]. The structured process mapping exercise focuses on how processes currently occur and includes identifying which personnel are involved, how long each step takes in a specific process, and potential failure points. The process maps are also serving as a baseline and can be compared to post-implementation process maps to understand how (and begin to understand why) the process may or may not have changed as a result of implementation activities (i.e., adaptations) [49, 50].

#### Data integration

We are utilizing findings from the key informant interviews, site visit observations, group process mapping, and group pre-mortem brainwriting to create site-specific summaries which will be used to adjust the adaptable components of each EBP. Following key informant interviews and the site visits, two trained SAGE staff members will complete a semi-structured debrief and templated memo to summarize the interview content [52]. Site profiles will then be generated from these summaries to inform the pre-implementation site visits and guide the next steps in implementation [52]. Additionally, a PRISM informed codebook will be developed to guide thematic analysis of interviews. Both the initial rapid content analysis and the more in-depth thematic analysis [53] will be used to gain a better understanding of the different organizational perspectives of the EBPs and organizational contexts to aid in creating a sustainable implementation plan tailored to the unique aspects of each site (Fig. 3).

### Phase 3: Active Implementation (6 months)

Following the pre-implementation assessment, the “Active Implementation” phase beings. To maximize fit between our chosen EBPs and each site, our active implementation strategies focus on facilitation, a flexible implementation strategy with a long track record of success for different EBPs in VA settings [26, 54–56]. It involves a partnership between external facilitators (i.e., SAGE staff) and the site implementation team, including front-line staff and operational leaders, who jointly plan and problem-solve issues related to implementation. We use the information gathered during pre-implementation assessments to inform which facilitation tools and approaches to use at each site. The current study will test the implementation strategies of facilitation, training and technical support, intervention tailoring, and audit and feedback.

#### Facilitation

Facilitation is the process of developing an interpersonal relationship focused on engaged problem-solving and support with the goal to implement an EBP within a given context [57]. Facilitation supports all aspects of implementation, but primarily Reach, Adoption, and Maintenance. For facilitation to achieve sustainable change, it is necessary to utilize a “highly partnered” strategy [58]. Facilitation is considered a multifaceted implementation strategy in that other implementation strategies are often used alongside and integrated with facilitation to support the implementation of an EBP. The SAGE project managers function as the external facilitators and support the implementation team for each EBP at a given site. The implementation team consists of a champion at each site to engage with key stakeholders, front-line staff, and older Veterans. For example, academic detailing pharmacists may act as champions for the EMPOWER intervention, whereas a surgeon and/or palliative care clinician may champion the Surgical Pause program, and a home-based primary care nurse practitioner may champion TAP or CAPABLE. Intervention facilitators will complete facilitation training offered by the VA Behavioral Health QUERI through the Implementation Facilitation hub before commencing facilitation activities.

#### Training and technical assistance

External SAGE facilitators will provide further support by utilizing the additional implementation strategies of training and technical assistance to the implementation teams. For example, training may take the form of education on the Best Case/Worst Case approach to goals of care conversations for the Surgical Pause [59], while TAP and CAPABLE have existing online training modules that are paired with in-person evaluation and demonstration of key skills (https://duo.online.drexel.edu/new-ways-for-better-days/; https://nursing.jhu.edu/faculty_research/research/projects/capable/). The Canadian Deprescribing Network has developed a series of professional videos plus toolkits for the implementation of EMPOWER (Deprescribing.org). Ongoing technical assistance is provided during weekly virtual “office hours” to answer questions related to any EBP or during implementation team calls. Technical assistance supports previous training efforts about each of the EBPs and focuses on ensuring that all relevant providers who adopt the EBP are able to deliver it with high fidelity.

#### Intervention tailoring

Intervention tailoring involves prespecifying changes able to be made to the EBP to support implementation in the local context. This allows for each of the four EBPs to have some degree of adaptability to the local context while still maintaining fidelity. As shown in Table 3, a priori, we defined which aspects of the EBPs are “core” (cannot be modified) and which are “adaptable” (can be tailored to fit local context) to support all RE-AIM outcomes [60]. Based on the findings from the pre-implementation phase, we will work with the EBP developers to help re-define the adaptable elements to tailor the interventions to each site.

#### Audit and feedback

Audit and feedback will provide data on performance and help sites with Reach, Adoption, and measuring preliminary evidence of Effectiveness (i.e., through run or control charts; [57, 61]). We will employ our experience in quality improvement to conduct rapid implementation and evaluation cycles to iteratively improve implementation strategies, particularly early in the Active Implementation phase. For example, we can review the VA medical record to evaluate what proportion of older surgical patients underwent frailty screening; or identify VA pharmacy fills for older Veterans who received the EMPOWER intervention. This approach will help the implementation team at each site to have a timely understanding of how implementation is going and together with the external facilitator problem solve to overcome identified barriers.

#### Data collection

During the active implementation phase, we will track audit-feedback data and collect a range of qualitative data (e.g., recordings of meetings, tracking implementation, qualitative interviews) to better describe and understand the “how” of implementation. Using a standardized tracking form, the evaluation team and external facilitators will track implementation activities such as implementation team meetings (i.e., facilitation), implementation office hours, and facilitation coaching office hours. The SAGE evaluation team will also conduct monthly semi-structured interviews with the EBP external facilitators to understand how the implementation process is progressing and describe lessons learned from each EBP and site. Likewise, the SAGE evaluation team will conduct interviews with the implementing clinicians and Veterans who have received the EBP—which will be used to understand the process of implementation. Last, the SAGE evaluation team will conduct follow-up site visit observations to develop current process maps and serve as a check on implementation fidelity.

### Phase 4: Consolidation (6 months)

In this consolidation phase, sites will have settled on a tailored implementation plan for delivery of the EBPs, and efforts will shift to promoting Maintenance of the EBP implementation (Fig. 3). While sites will receive technical assistance and audit-and-feedback reports during this phase, the goal is to facilitate the transfer of both technical and strategic skills to individuals at each site (e.g., ability to rapidly assess needs/resources, team management, and organizational change). SAGE-initiated support will decline and will transition to rely on implementation team-initiated and on an as-needed basis. The development of a sustainment plan and learning collaboratives will help support this transition.

#### Sustainment plan

The goal of the sustainment plan is to provide the implementation team with the resources and information needed to continue implementation indefinitely. During the first month of the consolidation phase, the external facilitator and implementation team will review the implementation step-by-step process using a worksheet to identify any changes needed to reflect the current implementation process. For example, the sustainment plan will include the following: an updated process map, monthly audit and feedback reports during active implementation, SAGE QUERI SharePoint link, information for ongoing EBP and Implementation Science office hours, and learning collaborative dates.

#### Learning collaboratives

Learning collaboratives across sites will involve monthly calls with the implementation teams working on a specific EBP who have completed the active implementation phase. The goals of the learning collaboratives include the following: (1) to create a space for implementation teams of a given EBP to have a space to engage with and learn from other sites and (2) to build a sustainable knowledge base for the given EBP across the VISN. Agendas for the learning collaborative will initially be driven by the implementation teams and will focus on peer support and problem solving to promote sustained EBP implementation. Learning collaboratives will support Adoption and Maintenance, as well as help us to track implementation adaptations [62].

#### Data collection

The SAGE study team will continue to use the implementation tracking process to document interactions with sites and/or implementation teams during the consolidation phase. While creating the sustainment guides, the external facilitator will ask the implementation team the following: “What would it take for this EBP to be sustained forever?”; “What resources—such as supports or information—are needed?”; “What additional resources can the SAGE team provide you?” This information will be recorded in a debrief form.

### Phase 5: Evaluation (12 months)

The study will conclude with the “Evaluation” phase. We will evaluate implementation using the RE-AIM framework as part of PRISM (Table 2), comparing Implementation as Usual to Active Implementation. We plan to start evaluation 6 months following the Consolidation phase at each site, to allow for 12 months for evaluation to provide longer-term information on Maintenance outcomes, a key gap in the literature [63, 64]. While our EBPs vary by specific clinical focus, location of intervention (e.g., clinic, home), personnel involved, and duration, they will all be part of a cohesive effort to shift care to be more consistent with an AFHS. Thus, we will examine each EBP in two ways. Across EBPs, we define our *primary implementation outcome* as Reach, since an AFHS is primarily defined by whether all older adults receive “Age-Friendly” care. Reach is defined as the proportion of Veterans eligible for each EBP that received that EBP during the implementation phase (Table 2). We will measure Reach aggregated to the cluster level across participating sites. We will also capture how frequently patients receive the first part of the intervention (i.e., screening or referral), but not the second part (i.e., full delivery of intervention). Our *primary effectiveness outcome* across EBPs is “facility-free days” or the number of days older Veterans remain alive and outside the hospital or nursing home (for post-acute or long-term care). We will evaluate this outcome among all Veterans eligible for at least one EBP as well as among EBP cohorts. Additionally, we will capture *secondary outco*mes specific to each EBP, using convergent mixed methods informed by the RE-AIM framework (Table 2). Maintenance will be measured across EBPs using our primary implementation outcome (Reach), analyzing whether the proportion of eligible Veterans enrolled in the EBPs is stable, improves, or declines by more than 10% at the time of Evaluation (1 year following Active Implementation).

#### Data sources

We plan to use a Residential History File approach to identify our primary effectiveness outcome (facility-free days). The Residential History File concatenates VA, Medicare (including Medicare Advantage), and Medicaid claims to describe longitudinal episodes of care for individual Veterans across VA and non-VA settings [65, 66]. This approach uses VA and Medicare data to identify acute care hospitalizations and the Minimum Data Set (collected for every post-acute and long-term care nursing home stay for Veterans in the VA, Medicare, and Medicaid files) to identify days in these facilities versus outside these facilities as well as mortality data.

#### Power analysis

Using previously described methods for power calculation in stepped-wedge trials [67], we used data from our nine VAMCs for Veterans age 65 and older to estimate within- and between-cluster variance of number of days in the community, with nine clusters, four time periods (including the Implementation as Usual period), a baseline mean of 306 days with standard deviation of 0.84, and between-cluster correlation of 11.6 days [67]. With these assumptions, we would be able to detect a change as small as 1.7 days in the community with 80% power with an average enrollment per VAMC of 611 Veterans across all years and across EBPs. Differences of 6 days are considered meaningful in the Centers for Medicare and Medicaid Services quality measures, suggesting we have power to detect meaningful changes in outcomes for these interventions [29].

#### Quantitative evaluation

We will use a generalized linear mixed model for each outcome that incorporates fixed effects for time and treatment phase and a random effect for the individual practice site. We will use a similar mixed model for our primary implementation outcome (Reach; [24, 54, 67, 68]. The treatment variable will be specified as a binary variable corresponding to study phase (pre- and post-Active Implementation). We will estimate the model using restricted maximum likelihood. First, we will examine the effect of treatment on the outcome in an unadjusted analysis. Second, we will conduct an analysis adjusting for patient characteristics: age, sex, race/ethnicity, comorbidities (Charlson comorbidity score; [69]), presence of cognitive impairment (diagnosis of dementia; [70]), or functional impairment (JEN frailty index score; [71]). Third, we will conduct an analysis that also includes practice-level characteristics that may be confounders (number of Veterans served by the site, presence of a surgical program, presence of a pharmacy, urban/rural location, whether site is VA-operated or contracted). Then, because we hypothesize that Veterans at highest risk may benefit most from an AFHS, we will repeat our analyses in subgroups of Veterans who are in a cohort of High-Risk, High-Need Veterans identified by the Geriatrics and Extended Care and the Geriatrics and Extended Care Data Analysis Center as being at highest risk of death and long-term institutionalization in a nursing home. Last, consistent with PRISM, we will also seek to identify the “how and why” of implementation testing for mediating site variables [72] such as the PACT implementation index score [73], elements of the VA All Employee Survey, baseline Strategic Analytics for Improvement and Learning (or SAIL) ratings, whether any leadership positions were vacant or changed during implementation, and Practice Environment Scale of the Nursing Work Index. For all analysis, we will cluster errors by site, potentially with cluster bootstrap procedure to account for a small number of clusters.

#### Economic evaluation business case

In the interest of improving the value of care along with effectiveness, we will conduct a budget impact analysis (BIA), which relates closely to a standard cost-effectiveness analysis but allows for the examination of shorter-term use of health care resources (over 1–3 years) as a value metric. A BIA approach was selected because it focuses exclusively on downstream costs of implementing 4Ms from the perspective of the VA. This outcome represents an ideal measure of value for our target population: all four EBPs are focused on increasing facility-free days and decreasing health care utilization. Further, this outcome can be fully and accurately measured with the data gathered during our Evaluation phase, allowing for accurate measurement of costs for both Active Implementation and Implementation as Usual phases. We will follow best practices for data reporting, including showing the cost of each intervention and total costs; costs or savings from downstream healthcare use for participants; and the implementation costs of the for EBPs [74]. For example, we will factor in the cost of time required of implementation staff through VA personnel data (e.g., wages, hours worked, additional staff needed) and project records, and capture changes in how processes are conducted (and potential time savings) across sites through comparison of pre-Implementation to post-Active Implementation process maps.

## Discussion

The overall goal of the SAGE QUERI program is to understand how best to implement and scale evidence-based practices aligned with the AFHS model within the VA. SAGE QUERI expands on prior Age-Friendly work by identifying four evidence-based interventions to address core tenets (4Ms) of the AFHS initiative, rigorously evaluating different implementation strategies, and assessing a clinically meaningful outcome across all four EBPs. The primary outcome—“facility free days”—is aligned with the goals of older adults to live independently in the community as they age, and with system and payer needs to reduce costs. We will test the effectiveness of implementation strategies of implementation as usual compared with external facilitation, training and technical assistance, intervention tailoring, and audit and feedback, while providing valuable information about the cost effectiveness of each intervention. Together, the SAGE QUERI program will provide an implementation model of AFHS-concordant care across an entire regional healthcare system, offering insights for replication across the VA in fulfillment of its operational commitment to becoming a nation-wide AHFS.

Notable strengths of the study methods and designs include our robust application of the PRISM framework, as well as our interdisciplinary mixed method approaches are designed to identify contextual factors to fit implementation strategies with local sites’ needs and preferences (Table 1). The framework also allows us to leverage the RE-AIM model to generate ongoing feedback during the study and analyze patient and implementation outcomes (Table 2). The use of a randomized type III hybrid effectiveness implementation study with a stepped wedge design will allow us to measure the impact of implementation of evidence-based practices aligned with the AFHS model on outcomes of older Veterans. Our quantitative “realist” evaluation will draw inferences about factors identified from the PRISM framework that may mediate implementation and effectiveness outcomes and quantify the costs relative to outcomes achieved. Likewise, the PRISM framework will inform the identification of internal and external implementation barriers and facilitators for each EBP at the staff, provider, and patient level through stakeholder interviews, observations, and focus groups. This will allow us to tailor and adapt EBPs to address barriers and optimize facilitators to fit the local contexts. Drawing on a wide range of qualitative approaches and will provide critical insight into best implementation practices and how these may vary by context.

Although all included EBPs have been previously tested in other settings, to our knowledge, none are part of routine care within VISN 4, or in the VA. As a result, we anticipate significant challenges to implementation and adaptation. For example, for TAP and CAPABLE, one anticipated challenge is first identifying where the EBPs will best fit within each site and who will implement the interventions, as there are a range of potential implementation settings (Table 3). In addition, the VA has robust home care programs (such as Home-Based Primary Care) that stakeholders may perceive as duplicating these efforts (or, alternatively, providing a useful platform for implementing them). Coordination of implementation may be challenging because it is difficult to engage stakeholders who cross multiple departments and settings [14]. Similarly, Surgical Pause requires close coordination between surgeons, palliative care clinicians, and pre-operative clinics that could include other staff (e.g., general internists, anesthesiologists), making implementation more complex.

A second set of potential challenges is that sites may not choose to implement all four EBPs, despite our hypothesis that implementing all four EBPs are likely to have synergistic effects across the 4Ms, and thus a greater impact on the primary clinical and implementation outcomes. To increase feasibility of adoption for each medical center, we have conceptualized and chosen EBPs that do not rely on a single site of practice (e.g., primary care or geriatrics departments) to be responsible for implementing all four interventions. Our goal is to have the EBPs employed in every clinical setting to which they apply (for example, Surgical Pause could be disseminated to inpatient surgery and to non-surgical procedural specialties) in the nature of the AFHS model. In addition, we strategically selected EBPs that align with VA national initiatives. Additionally, using preliminary data, we identified potential adaptations to the interventions that may overcome these barriers. For example, the TAP and CAPABLE models are led by occupational therapists (OTs). The VA employs OTs and also contracts with OTs employed by outside agencies to provide home-based care for Veterans. We thus have the option to implement these interventions using VA staff or by training contracted staff. These decisions have trade-offs but contribute to the rich contextual inquiry and are likely applicable trade-offs to other VA sites—such as generating knowledge to medical centers that differ in approaches for employing OTs.

Finally, we anticipate the VA—like all health systems—is dealing with the effects of the COVID-19 pandemic. We are already seeing disruptions due to the COVID-19 pandemic impacting our early pre-implementation data collection (e.g., limit ability to travel to sites) and anticipate it will impact the implementation phases (e.g., create competing priorities, reduce staff capacity, interfere with in-person intervention delivery). To overcome disruptions to data collection during the pre-implementation phase, we have incorporated ongoing pandemic-related challenges into our protocol with the knowledge that things will continue to change as variants/waves increase/decrease over time. For example, we created a modified hybrid approach that is allowing us to conduct site visits in person, virtually, or a mixture of both. Additionally, given potential limited capacity of SAGE team to travel, we anticipate the potential of being able to partner with sites’ local quality improvement departments, high-reliability leaders, and/or Whole Health staff (an established VA initiative centered around well-being of Veterans) to gather data and/or assist with the implementation process [20]. We will also monitor and track how the interventions are adapted to address ongoing disruptions due to the pandemic. It is clear that after 2 years of the COVID-19 pandemic, there is great need to identify novel to promote remote implementation strategies. In fact, we anticipate this will be an important value added to the field of implementation science as we spread these EBPs broadly during a pandemic.

## Anticipated contributions to practice

To our knowledge, this is the first large-scale randomized effort to implement AFHS aligned evidence-based practices in a national health care system. Given the pressing need to improve care delivery for older adults, we anticipate findings from this project will be timely and relevant. Deliverables include not just knowledge about how each EBP might be most successfully disseminated and implemented in the VA, but how they might affect novel outcomes (e.g., days at home), how each varies in terms of costs and changes in outcomes, and best practices for expansion beyond VISN 4 (even within a pandemic). Although this work was designed to be implemented internally within the VA for operational purposes, we plan to partner with the IHI and the John A. Hartford Foundation, as well as our Veteran Advisory Board and Technical Expert Panel, to create and deliver VA-specific toolkits hosted by IHI and the John A. Hartford Foundation on the national Age-Friendly initiative website, as well as on the websites of our national VA partners (GEC and National Surgical Office). We will also deliver VA-specific toolkits to be hosted by the Canadian Deprescribing Network and by TAP and CAPABLE directors and institutions. Each toolkit will contain a collection of materials including written documents, videos, and links aimed at educating VA leadership and clinicians about the AFHS model and implementation of each of the four EBPs. We will also incorporate materials designed for use in our Implementation as Usual phase, to assist future users in spreading the AFHS approach. Moreover, we will create “patient-facing” guides as a resource for Veterans and caregivers; emerging evidence suggests these resources can act as a “pull” mechanism for Dissemination as they create demand in the targeted populations [75]. Ultimately, we plan on reaching out to other VISNs with the goal of spreading these EBP nationally.

## Supplementary Information

**Additional file 1:** **Figure S1.** Study timeline.

## Data Availability

The datasets generated and/or analyzed during the current study are not publicly available due to identifying nature of patients and providers who participated in the qualitative data collection. Furthermore, the VA claim data has patient data that is not to be shared publicly. However, how data was collected and managed can be shared including the interview guides via the corresponding author on reasonable request.

## Abbreviations

ADL: Activities of Daily Living
AFHS: Age-Friendly Health System
BIA: Budget impact analysis
CPT: Current Procedural Terminology
EBP: Evidence-based practice
EMPOWER: Eliminating Medications Through Patient Ownership of End Results
IHI: Institute for Healthcare Improvement
OT: Occupational therapist
PRISM: Pragmatic Robust Implementation and Sustainability Model
QUERI: Quality Enhancement Research Initiative
RE-AIM: Reach Effectiveness-Adoption Implementation Maintenance
SAGE: Safer Aging through Geriatrics-Informed Evidence-Based Practices
SAIL: Strategic Analytics for Improvement and Learning
TAP: Tailored Activities Program
VA: Veterans Affairs
VAMC: VA medical centers
VASQIP: Veterans Affairs Surgical Quality Improvement Program
VISN: Veteran Integrated Service Network
