# Supplementary material for: Study protocol: Type III hybrid effectiveness-implementation study implementing Age-Friendly evidence-based practices in the VA to improve outcomes in older adults
Source: Implement Sci Commun. 2023 May 25;4:57. doi: 10.1186/s43058-023-00431-5 (PMC10209584; doi:10.1186/s43058-023-00431-5)

Additional file 1. Study Timeline – Over a five-year timeframe, each site (with three per cluster) will move through five consecutive phases: implementation as usual (IAU), pre-implementation (Pre-I), active implementation (Active I), Consolidation (Consol), and evaluation.


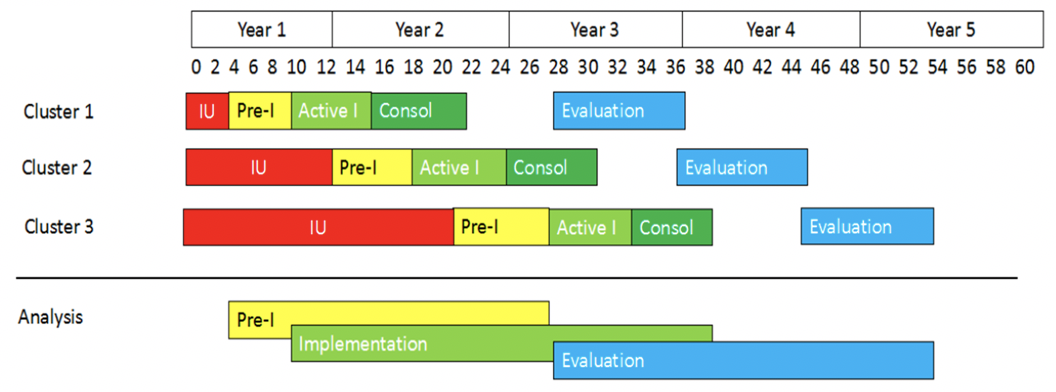

Supplement: Supplementary file 1 — Additional file 1: Figure S1. Study timeline. [file 43058_2023_431_MOESM1_ESM.docx]
